# Comparative study between manitol and sodium picosulfate with magnesium oxide solutions in the preparation for colonoscopy

**DOI:** 10.1590/0100-6991e-20222476-en

**Published:** 2022-05-05

**Authors:** CAROLINA MARTINS VISSOCI, GUSTAVO TRAVAGLIA SANTOS, ROBERTA PAIVA DUARTE, CRISTIANO GUIMARÃES DO AMARAL PINHEIRO, FERNANDO MARINHO MARQUES DA SILVA, VITOR PAIVA PIRES, JOÃO LUCAS FARIAS DO NASCIMENTO ROCHA, CASSIO SILVA COELHO, MARIA EDUARDA ROTTILI GOMES DE OLIVEIRA

**Affiliations:** 1 - Hospital Regional da Asa Norte, Unidade de Cirurgia Geral - Brasília - DF - Brasil

**Keywords:** Colonoscopy, Magnesium Oxide, Colon, Mannitol, Colonoscopia, Óxido de Magnésio, Colo, Manitol

## Abstract

This prospective, randomized and double-blind study aims to compare two different protocols used for bowel preparation in patients scheduled for colonoscopy. The protocols were composed by solutions of Mannitol or sodium picosulfate combined with magnesium oxide. Patients from the proctology outpatient clinic of the General Surgery Unit of the Regional Hospital of Asa Norte (HRAN) comprised the sample of this study. Both the patients and the colonoscopist had no prior knowledge of the substance used to prepare bowel, which was randomly distributed among the participants. Both protocols demonstrated good and similar results regarding the efficiency of colon preparation, although the review of literature shows a difference in favor of preparation made with Mannitol solution regarding the colon neatness during the exam. In line with the literature, patients who used Mannitol solution had more side effects, highlighting the significant difference found for vomiting and sleep impairment. The preparation with Sodium Picosulfate with Magnesium Oxide was significantly superior in relation to the ease of ingestion perceived by the patients.

## RESUMO

Estudo prospectivo, randomizado e duplo-cego com o objetivo de comparar dois protocolos diferentes utilizados para o preparo de cólon em pacientes que realizaram colonoscopia: solução de Manitol; e solução de Picossulfato de Sódio combinado com Óxido de Magnésio. Para avaliar qual protocolo proporciona melhores resultados, 90 pacientes do ambulatório de proctologia da Unidade de Cirurgia Geral do Hospital Regional da Asa Norte (HRAN) compuseram a amostra deste estudo. Tanto os pacientes quanto o colonoscopista não tiveram prévio conhecimento da substância utilizada para o preparo do cólon, a qual foi distribuída randomicamente entre os participantes. Os dois protocolos demonstraram bons e semelhantes resultados acerca da eficiência do preparo do cólon, embora a literatura estudada tenha demonstrado diferença significativa em favor do preparo feito com solução de Manitol em relação a limpeza do cólon durante o exame. Em consonância com a literatura, os pacientes que utilizaram solução de Manitol apresentaram mais efeitos colaterais, destacando-se a diferença significativa encontrada para vômito e distúrbios do sono. O preparo com Picossulfato de Sódio com Óxido de Magnésio foi relevantemente superior em relação à facilidade de ingestão percebida pelos pacientes.

## INTRODUCTION

Colonoscopy is well-established in the hospital environment as a standard procedure for the investigation and evaluation of colonic diseases and has been reinforced over the years. Its use in screening for intestinal neoplasms is ascertained in the literature. Colorectal cancer screening programs, which are widespread globally, have resulted in a dramatic increase in the number of colonoscopies performed in the last decade. Consequently, the search for better colon preparation regimens has intensified^1^ ^,^ ^2^. 

The safety and quality of this exam, which are related to diagnostic accuracy, are conditioned to the previous removal of fecal residues. These characteristics are directly dependent on the proper preparation of the colon, so that the organ is well visualized from the anal border to the ileocecal valve during the examination. In accordance with the patient’s needs and possibilities, the preparation aims to leave the colon completely clean for thorough analysis. In addition to detecting diseases, timely therapeutic interventions using colonoscopy also require proper colon cleansing^1^ ^-^ ^4^. 

Adequacy of preparation is important to ensure that existing injuries are identified. However, another aspect of great relevance is preventing the procedure from being prolonged beyond what is necessary or the proposed interventions from being canceled, which may result in diagnostic delay and a drop in the efficiency of the endoscopic resource^5^. 

The negative impact on the exam completion rate and the detection of adenomas caused by poor colon preparation has been repeatedly demonstrated in the literature, in addition to the increased risk of complications such as intestinal perforation. Different agents can be used for this preparation, which renders their comparison timely, seeking to assess whether there are more suitable ones^4^ ^-^ ^6^. 

The colon cleansing agent with an adequate profile should have reduced periods of ingestion and evacuation, while emptying all fecal material, solid or liquid, from the colon and rectum. It is also important to avoid gross or histological changes in the colonic mucosa, hydroelectrolytic variations, and discomfort by the patient^5^ ^,^ ^7^. 

 The tolerability of the preparation by the patient is of utmost importance, since the inability to complete the steps of the preparation leads to worse cleaning conditions for the examination, affecting patient compliance to screening programs^7^ ^,^ ^8^. In addition to patient tolerability, there is a concern in the literature about ease of administration, affordability, and how to prevent the preparation from resulting in the formation of explosive gases^3^. 

Over the years, most professionals have preferred anterograde colon preparation due to the quality of cleaning obtained and the patient’s comfort. The regimens currently available can be divided into two groups, consisting of large-volume, osmotically balanced substances, or low-volume, osmotically active ones. Larger volume preparations tend to be less tolerated^8^. 

Balancing the quality and acceptability of the preparation remains a challenge. In this context, two types of preparation were chosen for analysis and comparison in this work, those using Mannitol (Group M) and those using Sodium Picosulfate with Magnesium Oxide (Group P). The choice is due to the wide availability and use for the preparation of colonoscopy in clinical practice^8^. 

A member of the group of osmotic laxatives, mannitol is a derivative of mannose and is administered orally in a hypertonic solution that is not absorbed from the gastrointestinal tract. It tastes sweet and is administered diluted in juice for better tolerability. The patient needs to ingest a large volume, which can be divided over a long period of time (5-6h). However, a considerable number of patients have difficulty in ingesting the preparation, which can affect its result. Some studies have pointed to the risk of the fermentation of orally ingested mannose, causing the production of potentially explosive gases in the intestine, although the safety has been demonstrated in several studies. 

The combined preparation of sodium picosulfate with magnesium oxide is widely used, with good results documented in 85% of patients. Sodium picosulfate is a cathartic stimulant that is activated by colonic bacteria and acts primarily in the left colon. Magnesium oxide is an osmotic purgative that cleans the proximal colon^3^. 

Some authors also recommend a restrictive diet the day before to aid in the preparation, in addition to the combined use of bisacodyl to enhance the action of the other preparations. Bisacodyl is a contact laxative derived from diphenylmethane, with a hydragogue and anti-resorptive effect. It stimulates colonic peristalsis after hydrolysis in the mucosa of the large intestine, promoting accumulation of water and, consequently, of electrolytes in the colonic lumen^3^. 

Failure to prepare the colon for colonoscopy can result in failure to detect pathological lesions, in addition to cancellation and interruption of the procedure. The negative consequences of inadequate preparation are high for the health system, with significant harm to patient satisfaction. Failure of the procedure may result in delayed diagnosis and prolonged hospitalization^3^. 

Recent studies have shown that demographic variables and clinical characteristics can influence the quality outcome of colonoscopy. These findings highlight the importance for the continuity of the study on the preparation of colonoscopy, especially in the context of the public health service, which its inherent peculiarities, both structural and of patients. In the medical literature on the subject, the Brazilian population is rarely studied^9^. 

Being one of the most prevalent neoplasms in the world, colorectal cancer is the fourth leading cause of cancer-related death in both sexes combined. Among the risk factors for colorectal cancer are age (advanced) and the region of the globe, the most developed nations being those with the highest mortality rate. An increase of 80% in colorectal cancer is expected by the year 2030 due to demographic changes. South America underwent rapid epidemiological change caused by factors such as demographic transition, socioeconomic changes, and increased rates of overweight and obesity - with a dietary pattern based on sugars and saturated fats associated with a sedentary lifestyle. As a result, there was a decrease in the incidence of cancers related to infections in relation to cancers typically diagnosed in countries with better human development. Mortality in these cases is inversely related to early detection. Brazil presents an increase in the demand for preventive exams, such as colonoscopy. The impact of morbidity and mortality can be related to differences in infrastructure and access to health care and specialized exams^9^. 

Due to the status of colorectal cancer and the limited screening capacity in countries with transitioning populations, the implementation of comprehensive colorectal cancer monitoring is imperative. Quality data must be generated to improve knowledge on the subject^9^. 

## OBJECTIVES

 Given the importance of proper colon preparation for performing the colonoscopy exam, several studies have focused on the efficacy, safety, and tolerability of different forms of preparation. Under these circumstances, the present study aims to compare protocols based on two different solutions, the Sodium Picosulphate solution and the Mannitol one, as methods of colon preparation for the colonoscopy exam, to assess which solution provides better results.

 As a primary objective, we evaluated the quality of the preparation regarding cleanliness of the organ during the examination and adequate visualization according to the Boston scale. As a secondary objective, the aim was to evaluate the acceptability, the ease of use of the preparation, in addition to the occurrence of electrolyte imbalances and side effects.

## METHODS

This is a randomized, double-blinded study. The selected sample consists of patients scheduled to undergo colonoscopy in a proctology outpatient clinic of a public hospital, from August 2017 to March 2018.

We excluded patients who did not show up on the scheduled date, those who refused to participate, and those who were unable to follow the preparation instructions. 

Patients scheduled for colonoscopy received guidance on preparation in the week prior to the exam. The selection of the solution - Mannitol or Sodium Picosulfate with Magnesium Oxide - to be used in each patient was performed by simple randomization. The patients were not aware of the substance used, as the preparation was delivered without identification. The colonoscopy exam was routinely performed at 1:00 pm on Mondays, according to the service’s routine. 

All patients were previously instructed about the preparation and received a printed copy of the instructions when they received the preparation material, that is, in the week before the exam. On that occasion, doubts regarding the exam were clarified and the patients were invited to sign a consent form to participate in the study.

In both preparation methods, two bisacodyl tablets were used in the morning of the day before the exam, associated with a minimal residue diet. On the day of the exam, an exclusive liquid diet was recommended until 11 am, after which absolute fasting was instituted.

The mannitol preparation group (Group M) received a solution of 750mL of 20% Mannitol, to be diluted in 750mL of strained orange or lemon juice. Ingestion was divided into aliquots of approximately 200mL every 15 minutes and started at 6:00 am on the day of the exam.

For the preparation group with Sodium Picosulphate and Magnesium Oxide (Group P), the drug was presented in a sachet, to be diluted in 150mL of water for ingestion. In this group, the ingestion of two sachets of the solution was recommended. The first on the day before the exam, followed by the ingestion of at least five 250ml cups of clear liquid without residues. And the second, on the day of the exam, at 6:00 am, followed by the ingestion of at least three 250ml cups of clear liquids without residues.

Shortly before the exam, all patients answered a standard questionnaire, with information about demographic data, vital signs, exam indication, bowel habits, preparation tolerance, side effects, flavor, and other possible aspects related to the preparation. 

The colonoscopist was not aware of the preparation used by the patient and assessed the exam with a quality of preparation score according to the Boston scale, in addition to the description of the colonoscopic findings. 

For statistical analysis, we present numerical variables as mean and standard deviation, and categorical variables, as absolute and relative frequency. Comparison between groups was performed using the Mann-Whitney U, Chi-square, or Fisher’s exact tests. The significance level adopted was p≥0.05. All analyzes were performed using the Statistical Package for Social Sciences (SPSS) software, version 22.0.

## RESULTS

Ninety patients who underwent colonoscopy from March 2017 to July 2018 participated in this study. Of these, 55.6% belonged to Group M and 44.4%, to Group P. The continuous variables for the characterization of the sample are presented in the Table 1. It is noteworthy that there was no statistically significant difference between patients who received the different preparations for most variables, except for height (M: 1.6 ± 0.1; P: 1.7 ± 0.1; p=0.036) and heart rate (M: 87.3 ± 13.3; P: 82.4 ± 19.5; p=0.044).

**Table 1:** Sample characterization continuous variables.

	n	X ± PD
Age (years)	87	55.4 ± 13.8
Weight (Kg)	83	68.5 ± 14.3
Height (m)	82	1.6 ± 0.1
BMI (kg/m²)	81	25.9 ± 4.7
HR (bpm)	79	85 ± 14.7
BP Orthostatic		
Systolic	50	122.1 ± 22.1
Diastolic	50	81.6 ± 11.9
Mean	50	95.1 ± 13.7
BP Decubitus		
Systolic	78	124.5 ± 19.8
Diastolic	77	80.8 ± 12.1
Mean	77	95.3 ± 13.1

BMI: Body de Mass Index; HR: Heart Rate; BP: Blood Pressure.

**Table 2:** Sample characterization categorical variables.

	n	%
Gender		
Male	31	34.4
Female	59	65.5
Marital Status		
Single	21	25.9
Married	36	44.4
Widower	15	18.5
Divorced	9	11.1

Regarding the categorical variables, we observed no statistically significant differences between groups. The main indications for colonoscopy were digestive bleeding (35.6%), abdominal pain (16.7%), family history of cancer (15.6%), investigation of neoplastic focus (12.2%), and weight loss (11.1%).

As for previous comorbidities, the patients displayed systemic arterial hypertension (47.7%), diabetes mellitus (18.9%), unspecified arthropathy (5.5%), inflammatory bowel disease (4.4%), Chagas disease (3.3%), dyslipidemia (2.2%), asthma (2.2%), anemia (2.2%), psoriasis (1.1%), chronic pancreatitis (1.1%), chagasic megaesophagus (1.1%), chronic renal failure (1.1%), HIV (1.1%), hepatitis C (1.1%), gastritis (1.1%), fibromyalgia (1.1%), migraine (1.1%), gastroexophageal reflux disease (1.1%), dermatomyositis (1.1%), and unspecified cardiomyopathy (1.1%). In addition, the following previous operations were reported: gynecological (36.6%), cholecystectomy (11.1%), orthopedic (7.8%), mammoplasty (4.4%), urological (3.3%), perineoplasty (3.3%), exploratory laparotomy (3.3%), appendectomy (3.3%), inguinal herniorrhaphy (2.2%), esophagoplasty (2.2%), cataract correction (2.2%), left Hartmann colectomy (2.2%), myocardial revascularization (1.1%), intestinal transit reconstruction (1.1%), umbilical herniorrhaphy (1.1%), abdominal hernioplasty (1.1%), hemorrhoidectomy (1.1%), and unspecified varicose vein repair (1.1%).

When comparing the pattern of evacuation between groups, we found a significant difference in individuals in Group M, who exhibited a greater number of evacuations on the day of the exam (p<0.001). 

As for the difficulty in ingesting the preparations, we observed a statistically significant difference only in relation to the difficulty in ingesting the prescribed volume (p=0.008), with 27.7% of patients in Group M considering it difficult. Among Group P patients, this number was only 2.8%.

Side effects related to the colon preparation protocol were different between groups. There was a higher frequency of vomiting among individuals in Group M (M: 0.5 ± 1.3; P: 0.2 ± 0.8; p=0.047) and a higher incidence of sleep disturbance among those in Group P (M: 0.5 ± 1.2; P: 0.8 ± 1.5; p=0.022). There was no statistically significant difference for the other observed variables, such as abdominal pain, abdominal distension, and anal irritation.

Of the patients evaluated, 29 (36.7%) had already undergone colonoscopy before, and the following medications were used in the previous exam for preparation: mannitol (20%), Fleet enema^®^ (8%), bisacodyl (4%), and mannitol + lactulose (4%). In addition, 48% of respondents did not know which medication was used. When asked if they would take the same medication, most said they would (Group M: 93.3%; Group P: 90.0%; p=0.763). 

When analyzing the effectiveness of the protocol used for colon preparation, the result of the Boston Scale applied to both groups was considered satisfactory for most patients (Group M: 86.4%; Group P: 88.9%; p=0.734). 

 Additionally, serum sodium and potassium levels were collected immediately before colonoscopy, with no significant differences observed between groups (p>0.05) regarding variations in normality and electrolyte disturbances.

 In 17% of the colonoscopic examinations biopsies were performed as indicated by the colonoscopist. Tubular adenoma with low-grade dysplasia was found in 43.7%, adenocarcinoma in 18.7%, chronic colitis in 18.7%, and nonspecific chronic inflammatory process in 12.5%. Tubular adenoma with high-grade dysplasia, ileitis, chronic proctitis, chronic inflammatory bowel disease with microabscesses, or low-grade tubulovillous adenoma were observed in 6.2% of cases.

## DISCUSSION

The adequate evaluation and comparison of preparation methods for colonoscopy considered three factors. The first was the effectiveness of colon cleansing assessed using the Boston scale. The second was safety and side effects. The third was tolerability, which can influence adherence to the use of preparations by patients.

**Figure 1:**
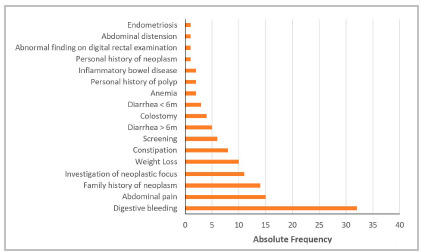
Indication for colonoscopy.

**Figure 2:**
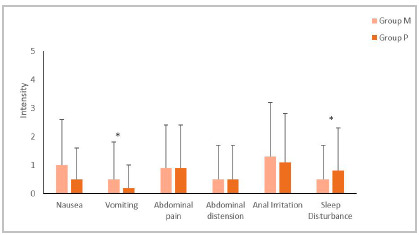
Comparison of side effects of patients taking the preparation in Group M (n=47) and in Group P (n=36). *Statistically significant difference.

**Figure 3:**
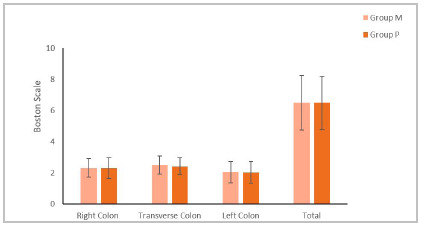
Comparison of the Boston Scale Result of patients in Group M and Group P.

Regarding the efficiency of the preparations, this study showed that both had good results and that they were very similar, with no statistically significant difference. This data is corroborated by the results of Miki et al. in a work carried out in the state of São Paulo in 2008^10^. However, both Müller et al., in the state of Rio Grande do Sul in 2007^3^, and Quaresma et al., in the state of Santa Catarina in 2018^11^, concluded that the use of mannitol would be more efficient than the use of association of sodium picosulfate and magnesium oxide. Both had statistical significance regarding colon cleansing, that is, absence of residues. 

Group M sustained more side effects than Group P in all symptoms surveyed, nausea, abdominal pain, abdominal distension, and anal irritation, however without statistical significance. Vomiting and sleep disturbance were also higher in Group M, with statistical significance, p=0.047 and 0.022, respectively. Despite this, we observed no health risks to the patients. These data are corroborated by the comparison studies between the two preparations mentioned above.

As for the difficulty of ingestion, Group P tolerated the flavor slightly better than Group M, but without relevant statistical significance. In addition, it was superior in ease of ingestion of the total volume, this time with statistical significance (p=0.008). This result agrees with the literature^1^ ^,^ ^2^ ^,^ ^11^. 

Despite the differences pointed out, most patients said they would take the same drug to undergo another exam in the future. Mike et al.^10^, Müller et al.^3^, and Quaresma et al.^11^ demonstrated the same result in their research. In disagreement with this study, none of the authors mentioned the administration of bisacodyl associated with the evaluated preparations.

It is worth emphasizing the limitation of having chosen a sequential convenience sample in this study, without calculating the sampling power to assess the representativeness of the results arising from the participants in this research.

## CONCLUSION

Both preparations showed good efficiency and were equivalent. In addition, they did not pose a risk to patients’ health of. However, the Group P preparation provided better patient comfort compared with the one of Group M. The conclusion of this work is similar to that of Miki et al.^10^, showing an equivalence between the preparations used when evaluated globally.

Even with the parity observed between the analyzed solutions, the continuous investigation of different forms of preparation remains valid, to foster the theoretical substrate on the subject, mainly in view of the growing importance of colorectal cancer screening, favoring the possible early identification of pathologies with proper colon cleansing.
